# A review on mechanisms and impacts of cold plasma treatment as a non‐thermal technology on food pigments

**DOI:** 10.1002/fsn3.3897

**Published:** 2023-12-26

**Authors:** Yousef Ramezan, Amir Kamkari, Armita Lashkari, Donya Moradi, Abbas Najafi Tabrizi

**Affiliations:** ^1^ Department of Food Science and Technology, Faculty of Pharmacy, Tehran Medical Sciences Islamic Azad University Tehran Iran; ^2^ Nutrition & Food Sciences Research Center, Tehran Medical Sciences Islamic Azad University Tehran Iran; ^3^ Department of Food Engineering, Faculty of Agriculture University of Tabriz Tabriz Iran; ^4^ Department of Food Science and Technology Islamic Azad University, Tehran North Branch Tehran Iran

**Keywords:** cold plasma, food color, non‐thermal technology, pigments

## Abstract

Food characteristics like appearance and color, which are delicate parameters during food processing, are important determinants of product acceptance because of the growing trend toward more diverse and healthier diets worldwide, as well as the increase in population and its effects on food consumption. Cold plasma (CP), as a novel technology, has marked a new trend in agriculture and food processing due to the various advantages of meeting both the physicochemical and nutritional characteristics of food products with minimal changes in physical, chemical, nutritional, and sensorial properties. CP processing has a positive impact on food quality, including the preservation of natural food pigments. This article describes the influence of CP on natural food pigments and color changes in vegetables and fruits. Attributes of natural pigments, such as carotenoids, chlorophyll, anthocyanin, betalain, and myoglobin, are presented. In addition, the characteristics and mechanisms of CP processes were studied, and the effect of CP on mentioned pigments was investigated in recent literature, showing that the use of CP technology led to better preservation of pigments, improving their preservation and extraction yield. While certain modest and undesirable changes in color are documented, overall, the exposure of most food items to CP resulted in minor loss and even beneficial influence on color. More study is needed since not all elements of CP treatment are currently understood. The negative and positive effects of CP on natural food pigments in various products are discussed in this review.

## Graphical abstract

Cold plasma (CP) is a novel non‐thermal food processing method. Color is one of the most important indicators of the quality of a product. Many studies reported that CP did not alter natural food pigments. The mechanisms of CP on the food color products are reported.

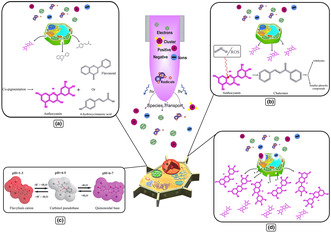

## 1 INTRODUCTION

Currently, both the population and the average level of educational attainment are rising. The demands of consumers are for a food supply that is free of chemical additives, safe, plentiful, and of excellent nutritional and sensory quality. Media evolution has made more information about the importance of this issue available to the public (Cheah et al., 2020; López et al., 2019). Nonetheless, according to numerous studies, conventional methods, such as thermal or chemical methods, are considered the root cause of nutritional loss in the food industry. Extending these procedures could alter attributes such as color, structure, and nutrient components to accomplish the desired result. The fact that traditional methods are not cost‐effective has also led producers to favor non‐thermal technologies (Bhatt et al., 2018; Mošovská et al., 2018).

Cold Plasma is a cutting‐edge, newly emerging technology that acts as a surface agent and has fewer degrading effects on food quality and nutritional value than conventional processes (Moradi et al., 2023; Pankaj et al., 2014). In the food industry, CP has been used for decontamination (Gavahian & Khaneghah, 2020; Ramezan et al., 2023; Wang et al., 2020), enzyme inactivation (Mir et al., 2020; Thirumdas & Annapure, 2020), and more recently CP has demonstrated significant dehydration potential (Ashtiani et al., 2020; Zhou et al., 2020). Although the exact mechanism of inactivation by plasma treatment is still unknown, the main proposed mechanism is the impact of reactive species like ROS and RNS induced by plasma on the cell wall and cell rupture as a result (Misra et al., 2016a). Color is one of the most important and influential organoleptic characteristics that immediately affect food quality, preference, and acceptability. Color is always associated with ripeness, vitality, and healthiness in the consumer's perception.

Consequently, a product's color may be one of the first factors taken into account when determining its acceptability (Mohamad et al., 2019; Neves et al., 2019). CP can be considered a viable alternative to prevalent thermal methods, which are known to cause inevitable changes during their processes. Several researchers reported no significant loss and a desirable effect on the color of the products; nevertheless, a few individuals reported slight unfavorable alterations after CP treatment. This study aims to provide a succinct depiction of the correlation between color and its acceptance among consumers, its impact on the taste of food, and the influence of CP treatment on food color products.

## 2 THE BASIS OF COLD PLASMA

Cold plasma is the fourth state of matter characterized by its gaseous nature and the presence of a net charge resulting from thorough or partial ionization induced by an external energy source, such as thermal, electrical, UV light, or electromagnetic sources (Gholamazad et al., 2022; Moradi et al., 2022). The production of reactive species, such as charged molecules, ions, free electrons, radicals, photons, and ionized molecules or atoms, is considered as a distinguishing feature of CP when an appropriate energy source is employed (Ekezie et al., 2017; Pedrow et al., 2020). When an electrical discharge is applied to a gas (or gas mixture), cold plasma is produced. Using air as a working gas produces a potent mixture of free radicals that includes UV photons as well as reactive nitrogen species (RNS) such as excited nitrogen (N_2_), atomic nitrogen (N), nitric oxide (NO), and nitric dioxide (NO_2_), reactive oxygen species (ROS) including atomic oxygen (O), superoxide anion (O_2_), singlet oxygen (O_2_), hydroxyl radicals (OH^.^), and ozone (O_3_). Moreover, when nitrogen serves as the system's inlet gas, a combination of nitrogen oxide (NO), atomic nitrogen (N), nitrogen dioxide (NO_2_), nitrate radical (NO_3_) (though it is more frequently found as nitrate ions, NO_3_^−^), nitrous oxide (N_2_O), dinitrogen trioxide (N_2_O_3_), and dinitrogen tetroxide (N_2_O_4_) is generated, highlighting that CP is typically produced at air pressure or decreased pressure, CP can be categorized into two distinct classifications: first, temperature‐based cold plasma, which encompasses high and low‐temperature cold plasma and secondly, pressure‐based cold plasma, which includes high‐pressure cold plasma, atmospheric pressure cold plasma, and low‐pressure cold plasma (Ahangari et al., 2021; Misra et al., 2016b). High temperature or thermal plasma is achieved when the temperature of electrons, atoms, molecular, negative, and positive species are tremendously high. In other words, thermal/equilibrium CP is a state of matter where the temperature of the electrons is about equivalent to that of the gases and ions. As pressure increases, more collisions occur, resulting in this state of matter. Energy is consequently split between the heavier species and the electrons (Peng et al., 2020). Non‐thermal/non‐equilibrium CP arises when the temperature of gases and ions is lower than the temperature of electrons. Dielectric barrier discharges (DBD), gliding arc discharges, corona discharges, and radiofrequency are among the various CP types that have the potential to function at atmospheric pressure. In order to prevent direct contact with CP and an electrical breakdown, DBD is generated between two electrodes that are separated by at least one dielectric barrier. As a result, even though CP is generated quickly using the DBD method, the temperature of the CP and the amount of reactive species that are produced are kept low (Pedrow et al., 2020; Sakudo et al., 2018; Teschke et al., 2005). The DBD CP are geometrically confined to the interelectrode gaps or the containment enclosure, whereas CP jets permit the ionized species to be launched outside. This would be the most important distinction between DBDs and CP jets in terms of application (Misra et al., 2019). Gliding arc discharges (GAD) are created in a reactor to produce thermal and non‐thermal CP through one or more electrodes operating at high potential differences. The combustion discharge will occur above the distribution orifice while an inlet gas is circulated between the electrodes, resulting in the formation of an arc discharge between the electrodes with the smallest surface area (Krupski & Stryczewska, 2020; Teschke et al., 2005). Due to the increasing channel between gas species in low‐pressure CP, the electron acceleration in the electric field predominates, which limits the background gas heat‐up brought on by heavy particle collisions. When electrons collide with certain plasma elements, such as air, primary reactions that release atomic and metastable oxygen and nitrogen are sparked. This is the beginning of a chemical reaction in cold plasma. Ozone, singlet O, or atomic O are produced as a result of secondary reactions that happen between neutral and ionic species (Magureanu et al., 2016; Scholtz et al., 2015). Low‐pressure CP will be produced by employing electromagnetic waves, such as radiofrequency and microwaves, at fast transmission rates. This technique uses a magnetron discharge to ionize the executive gas, which then absorbs the magnetron's radiation (Mehdizadeh, 2015; Pedrow et al., 2020). CP jet, gliding arc, dielectric barrier discharge, and low‐pressure CP are extensively studied in food processing.

## 3 COLOR PERCEPTION

**FIGURE 1:**
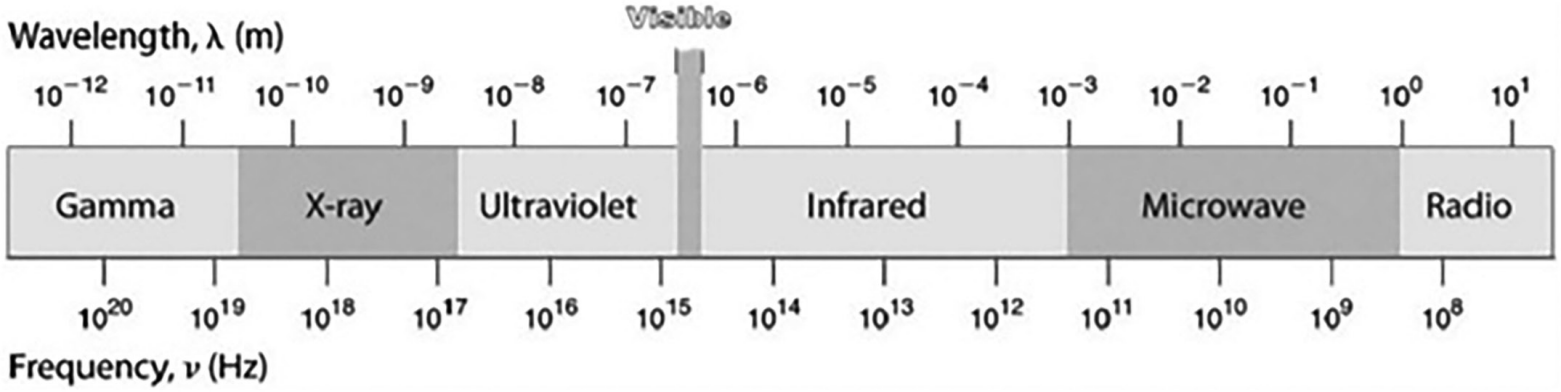
Wavelength range of human eyesight.

Perception occurs when the eye detects the waves' intensity and wavelength changes (De Valois & De Valois, 2001). The human eye can detect only a limited portion of the electromagnetic spectrum (radio waves, microwaves, infrared radiation, visible light, ultraviolet light, X‐rays, and gamma rays). Light reaches the human eye through the cornea and focuses on the retina, the innermost eye layer, which contains three types of light receptors: rods, cones, and intrinsically photosensitive retinal ganglion cells. The cornea is responsible for daytime vision since they are light‐adapted and sensitive to high‐intensity light. In other words, the rods are responsible for "scotopic" vision because of their sensitivity to dim light, yet their cell receptors are color‐blind (Grzybowski & Kupidura‐Majewski, 2019; Purves et al., 2001; Vohnsen, 2020; Witzel & Gegenfurtner, 2018). Intrinsically photosensitive retinal ganglion cells (ipRGCs) are light‐sensitive due to the presence of the light‐sensitive protein melanopsin and transmit light information to the brain through their axons (Do & Yau, 2010). The perception of color is attributed to the detection of visible light, which ranges in wavelength from 380 nm for the deepest visible violet to 780 nm for the deepest visible red (Figure 1). While all visible color to the human eye is a combination of the primary colors red, green, and blue, the wavelength of visible light varies from 380 nm for the deepest visible violet to 780 nm for the deepest visible red (Rodriguez‐Carmona & Barbur, 2017).

### 3.1 Food color and quality attributes relation

Color utilization in foods has a lengthy history, beginning in Egypt around 1500 BCE. A consumer's initial impression of a particular food product is primarily shaped by its appearance, and color plays a pivotal role in this regard. From the customers' perspective, food color is closely linked to perceptions of ripeness, freshness, and satisfaction, making it an early determinant of acceptability. This crucial organoleptic attribute directly influences the selection and acceptance of food products, rendering it one of the most significant factors for considering overall food acceptance. Foods that do not fall within the standard range of acceptable color are sometimes strongly rejected (Downham & Collins, 2000). Because color measurements are quick and easy to perform, and because they have been shown in one instance to have less variability than chemical analyses, color measurements may be a useful tool for plant breeders.

However, the cause of its effect seems to be learned associations rather than innate psychophysical traits. Research indicates that darker red solutions are perceived as sweeter than lighter‐colored solutions with the same sucrose concentration (Francis, 1995; Huang & Lu, 2016). Food colors are classified into synthetic, natural, and synthesized equally to the natural group. In the use of colors, specific rules regarding the maximum usable amounts, chemical properties, and purity have been set in the European Union. According to this statute, only colorants with defined acceptable daily intake and groups that have particular use permission can be used in foods, as the reaction between synthetic food additives and body cellular components may result in human health problems.

Our sensory perception of flavor derives from our expectations, which may be influenced by the relationship between color and sensory properties. For instance, the perceived odor becomes more intense with increasing darkness (Kemp & Gilbert, 1997; Zellner et al., 2018). In some investigations, the correlation between color and flavor has been reported, such that red corresponds to sweetness, and green corresponds to sourness (Spence et al., 2015). Also, the sweetness in beverages enhances by increasing the red color (Lavin & Lawless, 1998). As per *Goldstein's* theories, colors with a long wavelength can stimulate the senses and boost motivation. In contrast, short‐wavelength colors can make relaxation senses (Nakshian, 1964). Eventually, a number of studies have discovered a strong explanation for the impact of color on taste perception; nevertheless, cultural differences remain a significant factor that should not be disregarded. Indeed, one of the most crucial reactions that affect the color of fruits and vegetables is enzymatic browning, which leads to off‐flavor. Cold plasma treatment is one of the most effective mechanisms that decrease the rate of enzymatic‐browning reaction by reducing the activity of enzymes like polyphenol oxidase and pectin methyl esterase (Shankar et al., 2010).

Substitution of synthetic colorants with natural colorants has been challenging lately due to population growth and increasing food demand. The potential toxicity and associated hazards of synthetic pigments must be considered. These pigments are produced with greater color intensity and durability, with petroleum being the most used source. This approach is often more cost‐effective than the extraction and purification methods required for natural colorants. Since the health benefits of natural colorants, such as antioxidant, anti‐microbial, anti‐amyloid, and anti‐tumor properties, have led to increased consumer demand, their widespread application has been increased by industries.

The physicochemical stability of natural colorants depends on various factors such as pigment concentration, source of nutrition, purification methods, and enzyme activation. Also, the rate of pH, the intensity of light, the presence of oxygen, and the temperature are practical. Natural colors can be acquired from herbal, mineral, animal (including insects), and even microbial resources. The herbal origin allows the producers to extract pigments from a plant or a part of it, for instance, its leaves, roots, flowers, peels, or even fruits. In this regard, chemical interference risks with human health are minimized (Cortez et al., 2017; Martins et al., 2016; Mohamad et al., 2019; Neves et al., 2019; Özkan & Bilek, 2014; Rodriguez‐Amaya, 2019; Suparmi & Cahyono, 2015).

### 3.2 Effects of cold plasma on food pigments

As previously stated, color is one of the essential factors in accepting or refusing a product from the perspective of the consumer, so deleterious changes in the product's color due to improper processing or chemical changes have a direct impact on the consumer's acceptance and compliance, that is why, producers must be aware of proper processing technology (Wang et al., 2016). However, the application of CP aimed to enhance the antimicrobial efficacy of surfaces (Niemira, 2012; Sarangapani et al., 2016) despite being a surface agent, the utilization of CP in food processing has been extensively studied in various cases (Pankaj et al., 2014), focusing on its impact on food color and nutritional properties. In this regard, numerous researchers have reported that anthocyanins, carotenoids, betalain, and chlorophyll are the predominant pigments found in food. The impact of the CP on these pigments has been compiled in Table 1 and figures elaborated upon below.

**TABLE 1:** Summary of effects of CP processing on color of food products.

Samples	Method	Color changes	References
Blueberries	An in‐package high‐voltage dielectric barrier discharge CP Atmospheric air as a working gas Winding receives input at 230 V, 50 Hz and delivers a high‐voltage output in the range 0–120 kV Treatment times of 0 (control), 2, 5 min at 60 and 80 kV	Insignificant changes in color	(Sarangapani et al., 2017)
Strawberries	Single electrode non‐thermal atmospheric pressure CP jet used to generate CP activated water (PAW) A single electrode is connected to a 10‐kHz sinusoidal high‐voltage source with an 18‐kV peak‐to‐peak voltage 98% Ar and 2% O_2_ per volume, referred to as Ar/O_2_, used as working gas	No significant difference between L*, a*, and b* was observed The ∆E* values of most samples located in the range from 3 to 5, meanwhile 10‐PAW and 20‐PAW treated samples of day‐4 had higher ∆E* values than other samples, indicating a very distinct perceivable color compared to fresh strawberries	(Ma et al., 2015)
Mandarin	Microwave‐powered CP treatment The microwave generator produces a 2.45‐GHz wave discharge operated at a power level of 50–1000 W A 2.45‐GHz wave discharge operated at a power level of 50–1000 W	Insignificant changes	(Won et al., 2017)
Fresh‐cut melon	Air‐gas dielectric barrier discharge (DBD) generator three parallel pair electrodes 19 V and 3A power	Significant differences was observed only after 2 and 4 days between the 15 + 15 min treated and control samples Treated melon samples became darker and more translucent at the end of storge time	(Tappi et al., 2016)
Radish sprouts	Nitrogen‐cold plasma generated at 900 W and 667 Pa using a microwave generator (2.45 GHz) Variable power of 50–1000 W Treatment time of 0, 2, 5, 10, and 20 Nitrogen gas flowing at a rate of 1 L/min	Insignificant color change It did not cause significant oxidative stress	(Oh et al., 2017)
Bulk Romaine lettuce	Dielectric barrier discharge atmospheric cold plasma (DACP) The frequency range can be varied between 0 and 2400 Hz using the function generator Peak‐to‐peak voltage measured ranges from 0 to 76 kV	Insignificant color change	(Min et al., 2017)
Fresh fruit and vegetable slices (Carrot, Cucumber, pear)	Atmospheric‐pressure cold plasma The openings in the two electrodes were about 0.8 mm in diameter, and the depth of the exit opening is ∼1 mm Compressed air was used as the working gas at a flow rate of 5 standard liter per minute (slm) The operating output of the power supply was 30 milliampere (mA) at 500 V	Color parameters of the fruits and vegetables were found to be only minimally affected and considered acceptable ΔE* values of all three slices are in the small differences range The ΔE* value of the carrot slices changed somewhat more noticeably compared to the cucumber and pear slices No unappealing differences in the surface colors after CP treatment can be ascertained by visual inspection of the slices	(Wang et al., 2012)
Onion powder	Microwave‐powered cold plasma Low microwave density cold plasma treatment (LMCPT) or high microwave density cold plasma treatment Gas flow rate of 1 L/min CP generation powers were 400, 474, 650, 826, and 900 W Treatment times of 10, 14, 25, 36, 40 min	Insignificant changes in color parameters (L*a*b*) of HMCP‐treated samples CP treatment did not affect Browning Index (BI)	(Kim et al., 2017)
Shiitake mushroom	Dielectric barrier discharge (DBD) gas CP for direct sample treatment‐ 50 kV, 20 min Arc CP to produce CP activated water (PAW)‐ 50 kV, 20 min	There were no significant differences between the color values (L*, a*, and b*) of fresh (DC), soaked in distilled water (WC), and CP‐treated (PAW and DBD) samples 1 week of storage resulted in different ΔE values of samples: The PAW sample had the lowest ΔE values for both sides compared to that of the DC DBD CP treatment reduced the overall color variation of mushroom during storage as compared to that of the DC	(Gavahian et al., 2020)
Red chicory	Dielectric barrier discharge (DBD) CP source The distance between the fluid and the electrodes was 2 cm Treatment parameters were fixed at 19.15 V and 3.15 ± 0.5 A for 15 min.	No significant effect on color of red chicory	(Trevisani et al., 2017)
Tomatoes	Low‐pressure non‐thermal dielectric barrier discharge CP Voltage ranging of 1–11 kV at the frequency of 50 Hz At the exposure time of 4–6 min	Tomato color index (TI) was enhanced Nevertheless, in all the CP‐treated tomatoes, the lightness value decreased considerably	(Ranjitha Gracy et al., 2019)
Potato	Microwave cold plasma generated (CP) under low‐pressure conditions treatment At the microwave power of 500, 600, 800, or 900 W Treatment time of 0, 10, 20, 30, or 40 min	No change was observed in a* and b* values As the storage time after CP treatment increased, the L* values of all samples decreased, and the a* value was increased No significant difference in b* during storage Delaying the browning of potatoes	(Kang et al., 2019)
Bulk grape tomatoes	Dielectric barrier discharge atmospheric cold plasma (DACP) Voltages ranging from 28 to 34 kV Frequency ranging from 0.7 to 1.4 kHz Treatment times ranging from 1, 2, 3, 5 to 10 min	Grape tomatoes color did not change significantly Treatment for 3 min did not significantly affect the measured color	(Min et al., 2018)
Hyssop (*Hyssopus officinalis* L.)	Cold atmospheric plasma Dual power supply simultaneously, power 1: 0 to 25 kV, 50 Hz and power 2: 0 to 10 kV, 6 kHz In all experiments, power 1 ranged from 17 to 23 kV; and power 2 was held constant at 10 kV and 6 kHz Treatment times of 1, 5, and 10 min and the voltage values of 17, 20, and 23 kV The distance between the electrodes kept constant (0.5 cm) in all the experiments	In the case of a* a* value decreased with increasing both treatment time and voltage in digital still camera (DSC). The decreasing trend of a* value with increasing voltage level was observed at 10 min of treatment time for both the DSC and Hunter Lab In the case of b* Using the digital still camera, the b* value was greater than that of the control sample for all the treated samples except for 5 min and 20 kV treated sample For Hunter Lab, the b* value was greater than that of all the treated samples except for 5 min and 20 kV and 5 min treated sample and 23 kV In the case of L* Hunter Lab results show an increase for all the treated samples Using the DSC, only for 23 kV at 1 and 10 min an increase was observed	(Rezaei et al., 2020)
Peppercorns	UV‐cold plasma treatment Five UV‐C lamps were used for UV irradiation The output of 9.7–10.6 kV The CP frequency was fixed at 15 kHz	No significant changes in L*, a*, b*, and hue angle values was observed	(Bang et al., 2020)
Red pepper	Atmospheric pressure floating‐electrode dielectric‐barrier discharge CP Treatment time of 10, 20, and 30 min Generated using radiofrequency discharge of 13.56 MHz Powers ranges from 0 to 1000 W	After CP treatment for 20 min: L*(lightness) and a*(redness) significantly decreased b*(yellowness) significantly increased (*P ≤ 0.05*) under the exposure of CP at 20 min ∆E decreased	(Abdi et al., 2019)
Chili pepper (*Capsicum annuum* L.)	Atmospheric pressure plasma The samples were treated for 15, 30, 45, 60 s with gliding arc discharge CP equipment 3 L/min gas flow adjusted to a pulse frequency of 20 kHz, 750 W power consumption	No significant changes (in L*, a*, b*) Red pigment improved significantly Long pretreatment led to pigment loss	(Zhang, Zhong, et al., 2019)
Black pepper	1) Direct CP treatment with RF CP jet 2) Remote treatment (CAPP) with a microwave generated CP Argon working gas with a gas flow of 10 L/min Power of 30 W The samples were treated up to 15 min	Both methods did not significantly alter the quality parameters, only slight changes were observed	(Hertwig et al., 2015)
Siriguela Juice	Glow discharge CP 80 W and 50 kHz power supply at 5–15 min The nitrogen gas flow rate of 10–30 mL/min	Negligible color change Slightly enhance the color vivacity (+ΔC) Except the fifth assay, red color saturation increased (+a*) L* were higher than control samples b* slightly increased An increase in darkening color was not observed	(Paixão et al., 2019)
White grape juice	Non‐thermal high‐voltage atmospheric cold plasma (HVACP) The voltage input of 230 V, 60 Hz Samples were treated at 80 kV (peak‐to‐peak) for 1, 2, 3, and 4 min	No significant difference in the L*, an increase in a* and b* values were observed after treatment An increase in the chroma An insignificant decrease in Hue angle An increase in the browning of the juices depending on the treatment time Minor color change difference	(Pankaj et al., 2017)
Orange juice	High‐voltage atmospheric cold plasma Electric field voltages up to 90 with an electrical energy input voltage of 120 V (AC) at 60 Hz Applied for 30, 60, and 120 s	No significant color changed occurred in 120 s of treatment Treating orange juice with HVACP for 120 s causes slightly noticeable changes in ∆E	(Xu et al., 2017)
Tender coconut water (TCW)	Samples treated with ACP with both air and a mixture of: 65% O_2_, 30% CO_2_, 5% N_2_ (M65), as the working gas For 120 s at 90 kV	L* did not show major impacted for any treatment a* values shifted toward greener color Hunter b* value for TCW decreased significantly on ACP treatment with M65 ΔE values were not noticeable	(Mahnot et al., 2019)
Green coconut water	Atmospheric Cold plasma (ACP) processing A chamber containing two aluminum discs separated at a distance of 15 mm The frequencies applied were 200, 400, 550, and 730 Hz Voltages of 15 and 20 kV The processing time was 15 min for all treatments	Although ACP did not change color parameter but: Slight variations were detected in the individual color parameters, and ACP processing showed the smallest (ΔE > 2) compared to the ozone processing, but it was not noticeable	(Porto et al., 2020)
Blueberry juice	CP jet generated by argon and oxygen gas flow rate of 1.0 L/min Running at a constant voltage of 11 kV and frequency of 1000 Hz	Upward trend of L* and H* value, and downward trend of a*, b* and C* value, regarding with the increasing of treatment time or oxygen concentration after CP treatment	(Hou et al., 2019)
Cloudy apple juice	CP discharge: a) spark discharge b) glow discharge HV half‐bridge resonant inverter circuit with 20 kV max voltage and a variable frequency of 20–65 kHz.	Color significantly became greener (−a*) In all case, ΔE during storage was bigger than 3 L* only decreased after 1 min L* increased even maintained at extended periods and reached to the optimal at 5 min a* decreased with treatment time and its correlated with b* Juices with longer treatment time had bigger color change Non‐enzymatic browning increased right after the treatment but decreased during storage	(Illera et al., 2019)
Sea bass slices	High ‐voltage cold atmospheric plasma (HVCAP) Input voltage of 230 V at a frequency of 50 Hz, and output voltage controlled within 0–260 V With 2.0 cm working space	An increase in L* and a decrease in a* were obtained for with HVCAP as treatment time increased The control sample had the lowest L* value and highest a* value (p < 0.05) L* value was highest and a* value was lowest in the sample treated with HVCAP for 10 min	(Olatunde et al., 2019)
Chicken breast meat	In‐package dielectric barrier discharge (DBD) atmospheric cold plasma (70 kV, 0, 60, 180,300 s)	Comparing prepackaging to post‐treatment: There were no significant differences in a* and b* values of the CP‐treated chicken cutlets for any treatment time Significant increases in L* were found for 60, 180, and 300 s Comparing treatment time effect on the post‐treatment measurements to the no‐treatment control: Increasing treatment time to at least 18 s significantly increased L* Treatment times of 60 and 300 s significantly reduced b*	(Zhuang et al., 2019)
Pork meat	In‐package high‐voltage dielectric barrier discharge Two‐electrode, which distanced 40 mm Barrier discharge with high voltage at 85 kV for 60 s	DBD treatment did not affect L * values of fresh pork (P > 0.05) a*, b* values and metmyoglobin (MetMb) content were significantly changed after treatment in 60% MAP at day 12	(Huang et al., 2019)
Canned ground ham	Atmospheric pressure CP (APP) The CP was discharged at a voltage of 7 kV and a frequency of 25 kHz The total power dissipated into the CP was measured to be about 600 W The flow rate of ambient air was set as 1.67 × 10^−4^ m^3^/s	No significant differences were found in color values L*, a*, and b*	(Lee et al., 2018)
Chicken skin and breast fillet	Cold atmospheric pressure plasma jet High‐frequency voltage of 1 MHz, 2–3 kV Feed gas of argon or air Exposure times of 30, 60, 120 or 180 s Distances of CP jet nozzle to sample surface was 5, 8, or 12 mm	Insignificant impact on color values of chicken skin and breast without loss of product color quality Argon‐CP at 8 mm distance for 120 or 180 s, resulted in a significant increase of the ΔL* value a* and b* values remain constant An insignificant decrease in L* values of chicken skin and breast samples by up to 4.35 units following CP treatment Increasing the CP treatment time did not have a significant impact on ΔL* value	(Rossow et al., 2018)
Ready‐to‐eat ham	Dielectric barrier discharge (DBD) Frequency of 3500 Hz and a duty cycle of 70% The gap between the electrodes was maintained at ~5 mm Input power supply of a transformer with 300 W, produced a high pulsating voltage output of 0–28 kV with 0–2 mA current output	The L*and b* values of ham sample were not significantly changed compared to the untreated control sample The a* (red‐green) values of ham samples were significantly lower than untreated samples	(Yadav et al., 2019)
Raw pork during refrigerated storage	Cold plasma was generated with high‐voltage discharge in a vacuum chamber (diameter 250 mm, height 500 mm, vacuum 100 Pa) with laboratory pulsed CP reactor for 0, 2, 5, and 10 min The frequencies were between 20 and 100 kHz and 1.2 Kilovolt‐Ampere (kVA) reactive power	Significant changes in ∆E*, H, and C* were observed only for 10 min helium CP‐exposed samples Storage time decreased H about 30%. Also, slight differences were observed in the ∆E values during 14 days of storage Storage time significantly affected surface color of meat. H decreased about 30% during storage	(Ulbin‐Figlewicz & Jarmoluk, 2016)
Italian salami	Alternating current glow discharge CP with a voltage input of 46 V between two electrodes Samples placed in a vacuum chamber evacuated to 0.15 Pa with a voltage input of 46 V in 5‐ and 60‐min treatment time	A decrease in a* and b* values and, an increase in lightness (L* value) was observed	(Faria et al., 2020)
Fresh mackerel (*Scomber scombrus*)	Dielectric barrier discharge (DBD) The samples were treated in triplicate at two discrete voltages of 70 and 80 kV for different Treatment times of 1, 3, and 5 min	No clear trend observed However, a significant decrease in L* was observed	(Albertos et al., 2017a, 2017b)
Pork jerky	Dielectric barrier discharge (DBD) Frequency; 4 kHz Peak‐to‐peak voltage of 3.8 kV 1 cm distance between the sample surface and the electrode	With treatment time increment L*, a*, and C* increased gradually b* decreased as treatment time increased	(Yong et al., 2019)
Refrigerated chicken eggs	High‐voltage atmospheric cold plasma (HVACP) Voltage output of 0–130 kV at a frequency of 60 Hz The inoculated egg samples were treated at 85 kV for 5, 10 and 15 min in duplicates	No significant difference in yolk color and treated samples	(Wan et al., 2017)
Cheese slice	Dielectric barrier discharge (DBD) CP At 3.5 kV and a bipolar 50 kHz (low‐frequency range) square wave with a 50% duty cycle (*the ratio of time a load or circuit is ON compared to the time the load or circuit is OFF*.) Helium gas was used to generate CP at a fixed flow rate of 4 L/min Samples were treated under CP for 1 to 15 min	No visible change in the CP‐treated cheese slices was observed although the instrumental analysis showed a significant decrease in the L* value and an increase in the b* value	(Lee et al., 2012)
Milk	Low‐temperature CP Samples were taken at each time interval of 3, 6, 9, 12, 15 and 20 min	Milk samples did not show any important changes in color after 0, 3, 6, 9, 12, and 15 min, only a slight change was observed after 20 min The same color parameters L*, a*, and b* and ΔE existed between the control samples and treated samples ΔE for milk after 9 min of CP treatment with 9 kV was 0.25 while longer exposure to CP (20 min) caused slightly higher ΔE	(Gurol et al., 2012)
Unpeeled almonds	Cold atmospheric pressure plasma using a diffuse coplanar surface barrier discharge 400 (DCSBD) CP source Peak to peak voltage of 20 kV A frequency of 15 kHz Working gases were dry air, O_2_, N_2_, CO_2_ and a CO_2_/Ar mixture, consists of 90% CO_2_ + 10% Ar and	O_2_, CO_2_ and 90% CO_2_ + 10% Ar as a process gas had a minor impact on samples Air and N_2_ as a process gas had a major and significant color changes The treatment with air‐based CP has decreased the L* and b*‐values over 5 min N_2_ CP resulted in decreased L* and b* values until 2.5 min treatment Color changes were significantly observed only on treatments in which N_2_ was present either using only N_2_ or air	(Hertwig et al., 2017)
Tiger Nuts	CP‐activated water (PAW) prepared using an atmospheric cold plasma jet At an input power of 650 W For 3, 7, and 10 min The discharge gap between the surface of the CP nozzle and the water surface was 5 cm The inducer gas was compressed air at 0.2 MPa, flowing at a rate of 39 L/min	The L* values of all the sample groups and the chroma of the lone PAW‐washed samples did not change significantly The total color difference of sample extracts from PAW treated, significantly increased	(Muhammad et al., 2019)
Dried walnut kernels	Radiofrequency low‐pressure cold plasma (LPCP) treatment using normal air Input powers 20, 30, 40, and 50 W Treatment times 10, 15, and 20 min	All color parameters (L*, b*, ΔE, chroma, and hue angle) except a* significantly changed after LPCP treatment (*p < .05*) moisture content in the LPCP‐treated samples decreased compared to the control, which may be the most critical factor in discoloration of walnut kernels LPCP darkened the treated samples' color	(Ahangari et al., 2021)
Short and long‐grain rice flour	Dielectric barrier discharge Two aluminum electrodes of circular geometry (outer diameter = 158 mm) Two discrete voltages of 60 and 70 kV applied for 5 and 10 min	Untreated long‐grain rice showed higher L* Both rice types showed a significant effect on L*, a* and b* values 70 kV for 10 min showed highest, and 60 kV for 5 min showed lowest L* and b* value, respectively a* value decreased for both types of rice Greater differences were due to rice type	(Pal et al., 2016)

*Note*: The color parameters originally have asterisks in their formula.

#### 3.2.1 Carotenoids

Carotenoids are yellow, orange, or reddish fat‐soluble and hydrophobic pigments with an absorbing range of 300 nm up to 550 nm and pH stability of 4–6 (Hara et al., 2018). Carotenoids can be found bonded to proteins in photosystem I (PSI, or plastocyanin–ferredoxin oxidoreductase) and photosystem II (a water‐plastoquinone oxidoreductase multisubunit complex), which are located in the unstacked, stroma‐exposed membranes and in the stacked membranes of granule chloroplasts, respectively (Timlin et al., 2017). It is worth mentioning that photosystem I and photosystem II are the two multi‐protein complexes that contain the vital pigments to harvest photons and catalyze the primary photosynthetic endergonic reactions, producing high‐energy compounds by using light energy (Caffarri et al., 2014). Furthermore, even though the results have demonstrated that some carotenoids are oriented parallel to the hydrophobic membrane's surface, others have polar functional groups and connect across it with polar functional groups that are outside of it. However, the majority of carotenoids that are located inside the membrane are bound as parts of any pigment‐protein complexes in higher plants' photosynthetic apparatus in the photosynthetic apparatus (Dall'Osto et al., 2014; Jomova & Valko, 2013). More than 700 carotenoids have been identified naturally: β‐carotene, α‐carotene, β‐cryptoxanthin, lycopene, lutein, and zeaxanthin which are the principal dietary carotenoids in food.

Based on functional group, carotenoids can be classified into carotenes with pure hydrocarbon chains and xanthophylls with oxygen‐containing groups such as lutein. Non‐cyclic (lycopene), monocyclic (γ‐carotene), or dicyclic (α‐carotene and β‐carotene) carotenoids are the other categories that could be mentioned. Carotenoids possess a tetraterpenoid structure with mostly double trans‐conjugated bonds, varying from 3 to 15, except for bixin, crocetin, and food‐borne carotenoids (Damodaran et al., 2007; Eroglu & Harrison, 2013; Kiokias et al., 2016; Mariutti et al., 2018; Milani et al., 2017; Suparmi et al., 2020; Yahia et al., 2017). According to the results, since carotenoids are highly unsaturated with a centrally located conjugated double bond in their structure, they have been reported to be highly unstable against oxidation as well as isomerization.

Oxidation may transpire because of diverse factors, such as the modification of cell structure by rupturing the cell, augmenting its surface porosity, or exposure to light and humidity, in addition, elevated temperatures resulting from thermal processing and oxygen penetration through package permeability can lead to the formation of peroxides, metals, enzymes, and antioxidants, thereby promoting oxidation. Notably, non‐enzymatic oxidation leads to the oxidation of both Z and E isomers (Rodriguez & Rodriguez‐Amaya, 2009). Non‐thermal processing methods, such as high‐pressure or high‐intensity pulsed electric field, have been reported to cause minimal damage to food products (Barba et al., 2017; Fernandes et al., 2014; Rodriguez‐Amaya, 2015; Sigurdson et al., 2017).

##### 3.2.1.1 How cold plasma affects carotenoids

**FIGURE 2:**
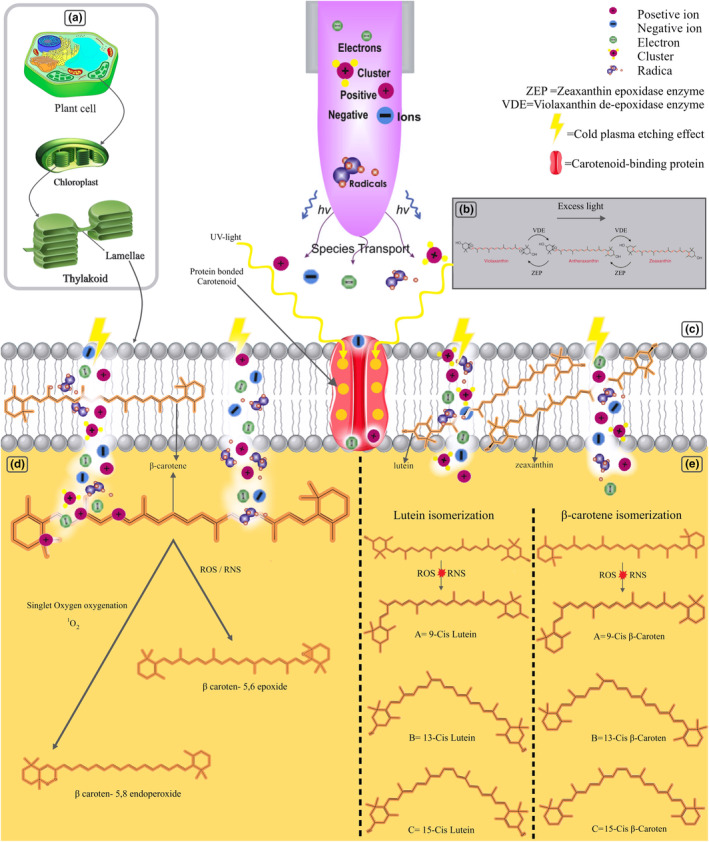
Effect of Cold Plasma on Carotenoid. (a). Carotenoid localization. (b). Photo‐oxidation of carotenoids by emitted UV light from Cold Plasma. (c). The outer space of the treated sample. (d). β‐carotene degradation due to the reactive oxygen species (ROS) interaction. (e). Lutein and β‐carotene isomerization as a result of ROS interaction.

CP causes changes in the structure of carotenoid pigments through different mechanisms, which is explained further. Carotenoids are stored in plastids of plants, so they are stable until the cell wall of plants is ruptured, after which the released carotenoids become sensitive to the environmental condition due to the carotenoids' unsaturated polyene chains interaction with CP‐generated oxidizing species that are rarely recognized in degradation carotenoids mechanism (Darvish et al., 2022). CP‐generated nitrogen and reactive species by changing the lipid‐soluble nature of cell wall leads to the creation of holes in the cell membrane which brings in leakage of the cell wall and increases the carotenoids release into the aqueous phase. When carotenoids are exposed to CP, alteration such as induced chain shortening or extension, hydrogenation, dehydrogenation, cyclization, double bound migration, and isomerization in the structural arrangement is inescapable (Figure 2).

Reactive species generated by CP can affect the color by releasing conjugated carotenoids' bound and/or breaking down crystalline carotenoid complexes and pectin complexes in the fruit cell wall. In addition, CP treatment reduces pH, which follows pigment‐protein complexes denaturation, and carotenoid content reduction regarding carotenoids' sensitivity to acidic conditions. On the other hand, since some of the carotenoids are bonded to specific binding sites in pigment‐protein complexes, they act as a protector for carotenoids; therefore, during CP treatment, the supramolecular structures of protein in the carotenoid protein complex break down by active chemical species of CP, so the carotenoid is released by pigment‐protein complexes denaturation (which is due to the pH reduction) and losing the protection effect (Figure 2) (Amorim et al., 2023; Pant et al., 2022; Umair et al., 2020). Hydrogen atoms can be added to unsaturated double bonds in the carotenoid molecule, resulting in the reduction of these double bonds and saturation of the carbon chain. This process leads to the formation of more stable and less reactive carotenoid derivatives. It has been declared that ring closure cyclization involves the formation of cyclic structures by closing one or more rings within the carotenoid molecule. Cold plasma could lead to the closure of the cycles in the carotenoid structure. It is when reactive species like singlet or triple oxygen are bonded that a dioxetane product is formed (Britton, 1976; Davies, 1969; Obileke et al., 2022). In a study, it has been reported that in long treatment times, highly reactive species, by producing sufficient energy, are prone to decompose fatty acids double bonds, which are more susceptible to peroxidative attack by reactive oxygen species (ROS). For instance, unsaturated fatty acids in milk were dramatically reduced with a parallel increase in primary oxidation products (Afshar et al., 2022; Korachi et al., 2015).

CP treatment on carrots, with different voltages and time values, was evaluated, and no significant changes were observed (Bermúdez‐Aguirre et al., 2013). For orange juice (Dasan & Boyaci, 2018), a* value decreased after 30 s. However, there were only slight differences after longer treatments. Regarding treatment time, the b* value showed a statistically significant but slight downward trend, while ΔE remained the same during the treatment, and no statistically significant differences (*p > .05*) were observed. In another study, ΔE of the carrot slices changed more noticeably due to greater surface carotene oxidation, despite being acceptable and having the slightest change (Wang et al., 2012). In addition, a corona discharge gas–liquid type was designed to investigate the effect of CP on pumpkin puree solutions under atmospheric pressure. Generally, significant variation occurred between 5 and 15 min of exposure. L* decreased marginally after the treatment; when a sample is sterilized, L* decreases more significantly.

Also, a* presented a significant loss in an untreated sample, and b* was slightly higher after the treatment time (Dasan & Boyaci, 2018). A mixed juice mixture of 20% carrot juice and 80% orange juice was exposed to a DBD system for 5, 15, and 30 s.

The most noticeable change in the control samples was the reduction of L* for 15 s of CP exposure. However, after 30 s, a* and b* decreased significantly without adverse effects on the quality. After a treatment time of 30 s, the highest increase in brightness value was achieved. In this study, the total color difference increased with treatment time duration. Moreover, a statistically significant increase in hue angle was observed after the first and most prolonged treatment. The color of the juice appears to be more apparent after 30 s of treatment, but two other shorter treatments cause an increasing trend in color saturation (Wang et al., 2012). The results of a study on the effect of CP on paprika color revealed a significant change in sample color, as the a* values of treated samples decreased, indicating a reduction in the product's redness.

Since carotenoids give paprika its characteristic red hue, increasing the spice's yellow hue results in a corresponding rise in L* and b* (Santos Jr et al., 2018). The oxidation of carotenoids and reduction of their redness is believed to be caused by the formation of active species such as HNO_2_, likely due to the CP treatment (Vukić et al., 2018). Darvish et al. (2022) investigated the effect of low‐pressure CP (LPCP) on decontamination and quality attributes of Saffron (*Crocus sativus* L.). All LPCP‐treated samples exhibited a substantially greater total color difference (∆E) than the control sample, which was deemed a favorable phenomenon for saffron (*p < .05*). Moreover, it was reported that aqueous saffron extracts prepared in boiling water had created more color in the treated samples than in the control ones. In this study, it was determined that carotenoids are the primary pigments of saffron and that the presence of conjugated double bonds is necessary to create a different color, noting that a more significant number of conjugated double bonds results in a higher absorption maximum (λ_max_), which can increase the red color intensity of the product (Darvish et al., 2022).

#### 3.2.2 Chlorophyll

Chlorophyll is a fat‐soluble pigment derivative from pyrroles characterized by a centralized magnesium ion centered in a porphyrin ring consisting of five nitrogen bonded carbon‐ring with a phytol chain, creating nearly all visible colors spectrum. The primary source of chlorophyll in rich‐chlorophyll plants is the finely fragmented leaf blades. Chlorophyll a and b are the two main chlorophyll varieties in food, where chlorophyll a contains a methyl bond and chlorophyll b contains –CHO in the seventh position. The pick absorbance of chlorophyll a and b are 430–664 and 460–647 nm, respectively (Alberts, 2017; Croft & Chen, 2017; Hanelt et al., 2003). Since chlorophyll is a hydrophobic pigment due to its phytol chain and by mentioning that the chlorophyllase enzyme is deactivated as the temperature rises at 100°C, chlorophyllase (EC 3.1.1.14) activity, as a phytol catalyzer agent, lead to the formation of a water‐soluble chlorophyllide with a light green color (Brazaitytė et al., 2019; Ladanyia & Ladaniya, 2010; Willows et al., 2013).

Chlorophyll is highly sensitive to heat, enzymes, oxygen, and pH, and its stability in pH ranges between 7 and 9 and degrades from 3.5 to 5 (Andrés‐Bello et al., 2013; Koca et al., 2007) as chlorophyll is subjection to weak acid and displacing Mg^2+^ ion with hydrogen and forming pheophytin, an olive‐green color derivates, while the displacement of Mg^2+^ with Cu produces a Cu‐chlorophyll complex that is stable against acidic conditions with a desirable green color (Beale, 2009; Humphrey, 2004; Motilva & Romero, 2010; Yilmaz & Gökmen, 2016).

##### 3.2.2.1 How cold plasma affects chlorophyll

There are four theories for the chlorophyll degradations mechanism, including type I: decomposition and enzyme denaturation, which slows down the chlorophyll catabolism operated by enzymes like chlorophyllase and Mg‐dechelatase.

**FIGURE 3:**
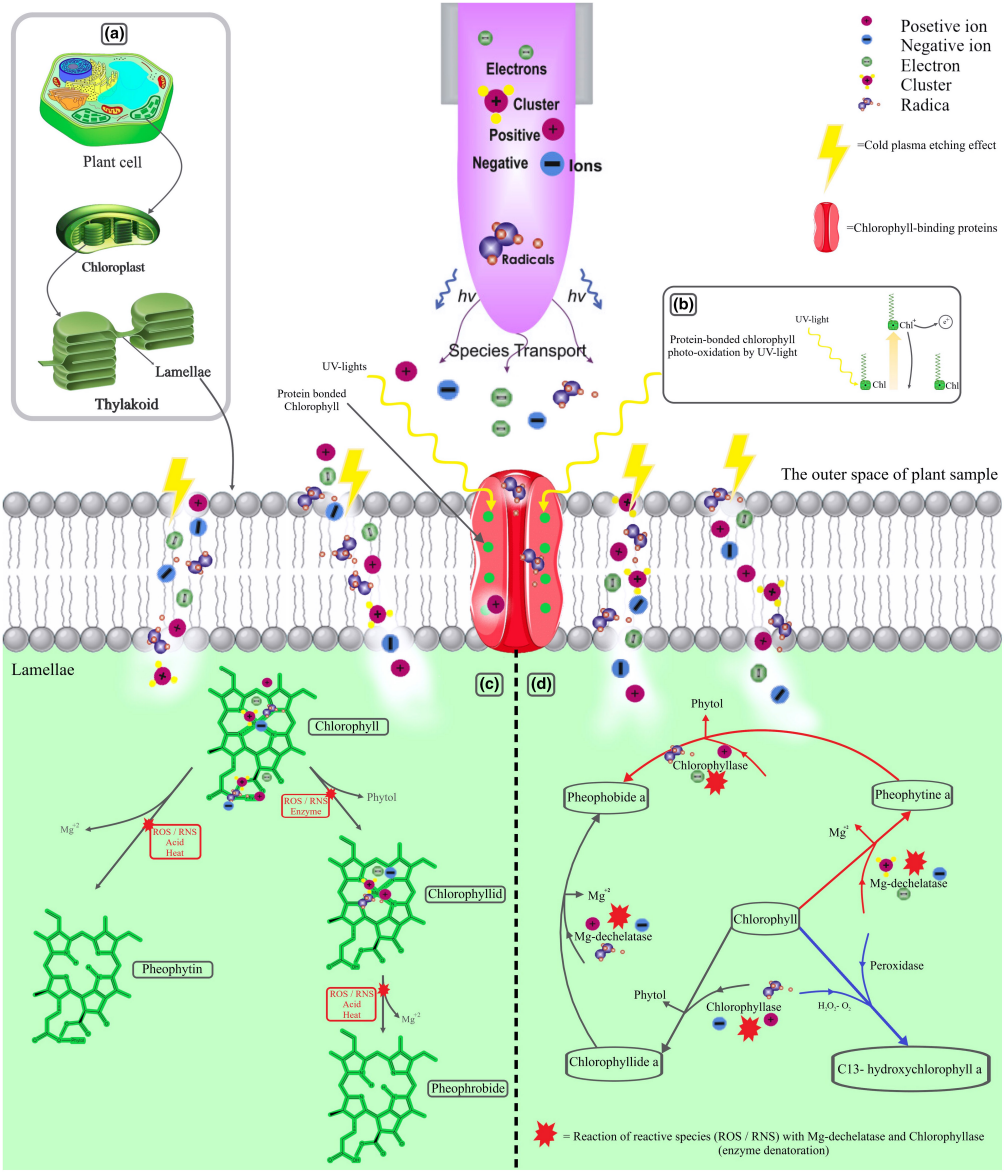
Effect of Cold Plasma on chlorophyll. (a). Chlorophyll localization. (b). Photo‐oxidation of chlorophyll by emitted UV light from Cold Plasma. (c). Effect of pH reduction on chlorophyll due to the plasma treatment. (d). Chlorophyll catabolism deceleration due to the decomposition and denaturation of operated enzymes like chlorophyllase and Mg‐dechelatase.

Type II: decomposition and degradation of the pigment by CP generated ROS; Type III: Because CP treatment causes a pH reduction when chlorophyll is subjected to a CP treatment, replacing Mg^2+^ by H^+^ through occurs, which results in the production of brown‐colored pheophytin and eventually pH reduction. Chlorophyll is more degraded by applying oxygen as a working gas than using nitrogen due to the higher reactivity of ROS than RNS in reaction with chlorophyll. Generally, the oxygen radicals generated by CP affect pigments in two ways, including pigment oxidation by oxygen radicals and protein denaturation, which decreases enzymes involved in chlorophyll catabolism. The last theory (type IV) is the possibility of photon and UV‐light radiation during CP treatment, which brings in the photooxidation of chlorophyll (Amorim et al., 2023; Nowacka et al., 2021) (Figure 3).

In a study conducted by Kashfi et al. (2020), the effect of low‐pressure CP on peppermint was investigated, and it was reported that as the applied power increased from 20 to 60 W. That means the treated samples got darkened, possibly due to the polyphenol oxidase activity. In this case, the parameter a* has been changed from −6.37 (untreated control) to −4.18 (60 W) by increasing the power while the untreated control sample was greener than the treated samples. In general, low‐pressure CP caused an increase in ΔE and decreased chroma and hue angle compared to the untreated control sample (Kashfi et al., 2020). The impact of atmospheric pressure CP on Romaine lettuce (*Lactuca sativa L. var. longifolia*) was investigated in another study, and a significant increase in a* after 3 min of treatment was stated. Meanwhile, the hue angle increased significantly after 7 min of treatment (Bermúdez‐Aguirre et al., 2013). Furthermore, kiwifruit was treated with DBD‐CP at atmospheric conditions, and the lightness, hue angle, and chroma value of the treated samples were decreased during storage time. However, a less darkened product resulted immediately after the CP process; in contrast, no significant changes were observed after the treatment among the control and treated samples (Ramazzina et al., 2015).

Nevertheless, this investigation showed that CP treatment reduced the reduction rate of chlorophyll during the storage; approximately 15% of chlorophyll was decreased instantly after the treatment with CP. The reason for this decline may be the type III fracture mechanism. In this mechanism, chlorophyll is oxidized by radicals generated in CP or from the fracture of surface components. Since CP is responsible for the partial denaturation of proteins (Kashfi et al., 2020; Tappi et al., 2014), the catabolism of produced chlorophyll by chlorophyllase and magnesium dechelatase is reduced during storage. In another study effect of non‐equilibrium atmospheric DBD was investigated on baby kale. Firstly, it was indicated that after 300 s of treatment, the hue angle, a*, and L* of the cut leaves were not negatively affected.

Moreover, polyphenol oxidase and peroxidase were prevented partially, while browning was thoroughly prevented. Secondly, in the whole leaves samples, L* increased and a* decreased insignificantly, and the browning index increased insignificantly by increasing treatment time (Shah et al., 2019). Also, the vivid hue may have contributed to the color bleaching. In another study, a CP torch device by a microwave source of 2.45 GHz was used for fresh chlorophyll‐containing green apples and cucumbers processing. According to the findings, 2.5 min of treatment on cucumber samples results in a lower L*. 48 h of storage and treatment periods of 5 and 10 min did not have a discernible impact on the a* and b* values. Only on days 0 and 2 of storage b* and L* were somewhat reduced for treated apple samples. Also, there was no discernible alteration in the hue of the green; it only seemed somewhat brighter (Baier et al., 2015).

By bombarding leafy vegetables in CP treatment, it should be mentioned that the active species, besides the possible destructive effects, often affects the primary layer of vegetables containing cuticle and wax (Grzegorzewski et al., 2010, 2011). However, regarding non‐leafy vegetables and fruits, it must be emphasized that carrot has no outer wax layer, and CP treatment causes damage to their primary cells. Therefore, CP causes the leakage of its internal contents into outer space and more color changes. Due to the contradictions about the impact of various CP sources on the surface of fruits, vegetables, and chlorophyll, the effects of all CP methods during storage should be evaluated for a more precise examination.

#### 3.2.3 Anthocyanins

With more than 700 distinct structural variations, anthocyanins, which range in color from red to blue and purple, are one of the most important components of water‐soluble colorants (Coultate, 2009; Zhang, Celli, & Brooks, 2019). Anthocyanin is the secondary plant metabolites which is chemically a class of polyphenols and belongs to flavonoids. From a structural perspective, anthocyanins are flavylium ion derivatives characterized by their polyhydroxy or polymethoxy derivatives of 2‐phenyl benzo‐pyrylium.

Anthocyanins are typically structured as 3,5,7‐trihydroxyflavilium chloride. Regarding their glycosidic structure, the non‐sugary component comprises aglycone or anthocyanidin, while the sugary component is most composed of glucose, galactose, or rhamnose. Typically, the sugar moiety bonds with either the hydroxyl group located at the third position on the C‐ring or the fifth position on the A‐ring. Since anthocyanins are sensitive to pH, they may act as a pH indicator and form a different structure like red flavylium cations in pH less than 2 and increased pH up to 4 in blue quinoidal bases (DeMan et al., 1999; Rodriguez‐Amaya, 2016; Waring & Hallas, 2013).

Pelargonidin (in strawberries), cyaniding (in apples and peaches), delphinidin (in oranges), peonidin (in cherries), cyanidin (the most abundant of all), and petunidin and malvidin (in grapes) are the most extensive and essential anthocyanins in the human diet and health. Cyanidin and its monomethylated product, peonidin, were equally distributed in the exocarp and endocarp. In contrast, delphinidin and its methylated products, petunidin, and malvidin, were more abundant in the exocarp than in the endocarp (Yoshimura et al., 2012). The greatest intensity in the blue color (hydroxyl group) and red color (methoxy predominates) belongs to delphinidin and malvidin, respectively. In addition to pH, anthocyanins are affected by heat (the most sensitive pigment), light, oxygen, solvents, and the presence of enzymes, proteins, and metallic ions (Jackman & Smith, 1996; Li et al., 2017; Smeriglio et al., 2016).

##### 3.2.3.1 How cold plasma affects anthocyanin

**FIGURE 4:**
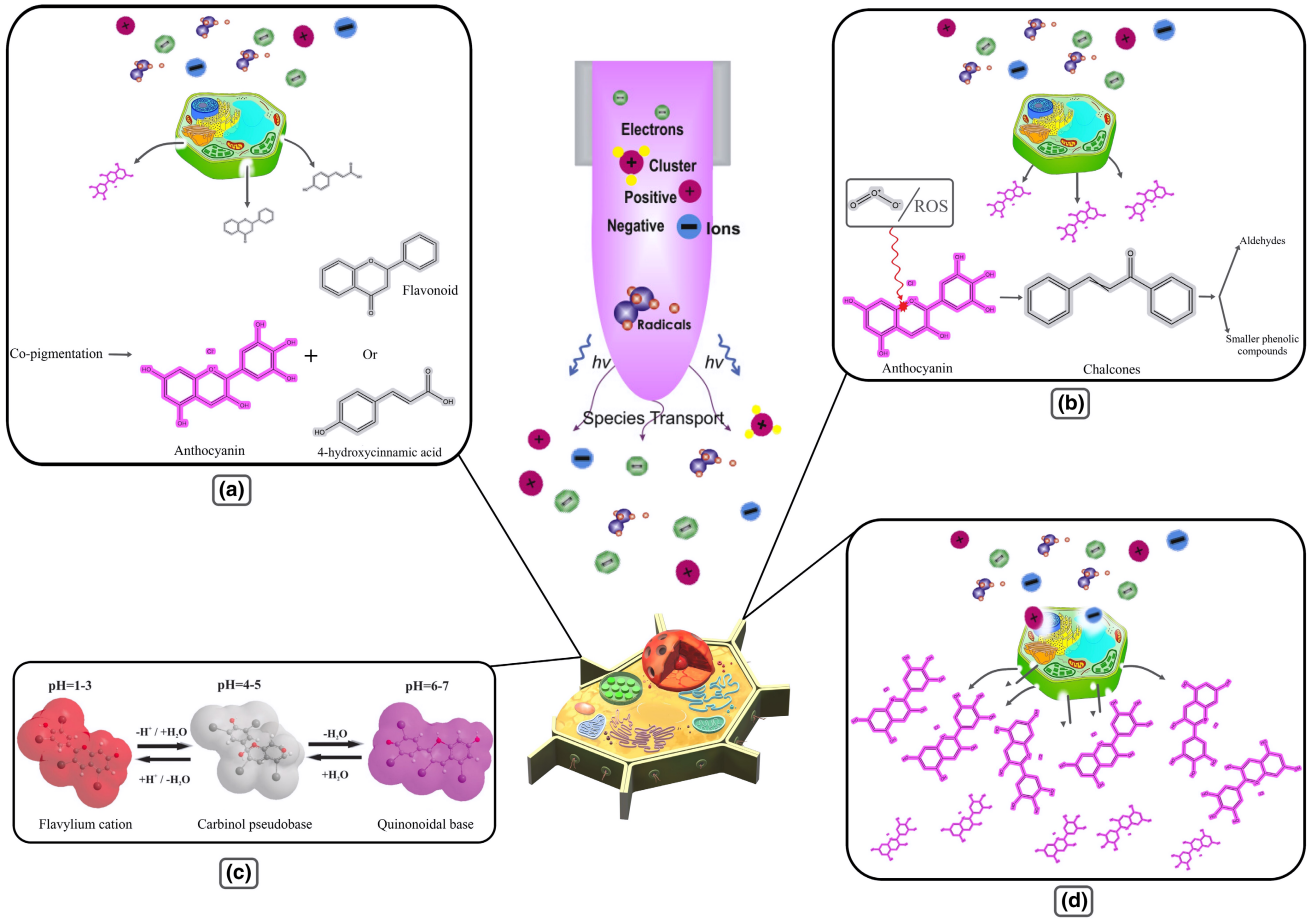
Effect of Cold Plasma on anthocyanin. (a). Co‐pigmentations of anthocyanin with extracted flavonoids and 4‐hydroxycinnamic acid as a result of membrane rapture. (b). Formation of chalcone as a result of ozone (as working gas)/ROS reaction with pyrylium ring and decomposition to aldehyde and phenolic compound. (c). Structural alteration of anthocyanins resulting from the change in pH caused by cold plasma treatment. (d). Increasing anthocyanin extraction as a result of membrane rapture.

The increase in anthocyanin content can be due to the improvement in their extraction due to the fruit cell structure disorder in the CP treatment process. CP causes a partial or thorough disorder in the cell membrane that causes the dispersion of internal contents into outer space (Kobzev et al., 2013). The disruption of the plant cell wall facilitates the penetration of solvents, thereby enhancing the matter transfer and extraction of polyphenols, resulting in greater anthocyanins extraction compared to conventional techniques (Landbo & Meyer, 2001; Tiwari et al., 2009). However, CP treatment can increase the colored anthocyanins or other compounds like flavonoids, alkaloids, amino acids, polysaccharides, and organic acids (Brouillard et al., 1989), the intensity of L* and a* values does not change the color; this can be due to co‐pigmentations that often occur in the liquid medium or by the presence of water molecules (Figure 4) (Fischer et al., 2013). It has been suggested that the Argon ion as a working gas, in conjunction with active oxygen species such as OH, O_3_, and O_2_, can disrupt the upper epiderm layer of plants and facilitate the distribution of flavonoids, phenolic, and other cellular components from the central vacuole (Grzegorzewski et al., 2011).

On the other hand, cell wall rupture during CP treatment facilitates better interaction between solvent and anthocyanin. The stability of anthocyanins depends on pH, which is more stable in low pH (acidic conditions). Anthocyanins are stored in the vacuoles of plants, and CP treatment destroys the vacuoles membrane and the cell wall. Therefore, anthocyanin is released into the external environment with higher pH, leading to a significant loss. Also, it has been concluded that ozone (applied gas) or ROS generated by CP treatment reacts with the pyrylium ring of anthocyanin and results in the formation of chalcones, which decomposes into aldehydes and smaller phenolic compounds (Amorim et al., 2023; Sui et al., 2014). Since CP treatment increases the extraction of flavonoids, alkaloids, amino acids, polysaccharides, and organic acids (such as hydroxycinnamic acid), Co‐pigmentation of anthocyanins with flavonoids and hydroxycinnamic acid, which are the most influential compounds, increases anthocyanin endurance. It should be mentioned that the possibility of Co‐pigmentation formation and anthocyanins endurance get increased by increasing CP treatment (Bąkowska et al., 2003; Davies & Mazza, 1993; Mazza & Brouillard, 1990). Co‐pigments enhance the durability of anthocyanins and the color variations caused by a shift in the spectral properties of chromophore groups. This effect increases the absorption property and transfers the visible spectrum's maximum wavelength to longer wavelengths, causing anthocyanin solutions to appear bluer and more intensely colored. The chemical structure and stability of anthocyanins may also contribute to the rise in their abundance. Hence, previous studies highlight the relationship between CP treatment and an increase in the durability of phenols and glycoside groups; the glycosidic group of anthocyanins may be responsible for this durability. According to reports, glycoside components such as quercetin‐4'‐O monoglucoside and quercetin‐3,4'‐O‐diglucoside degrade more slowly than the corresponding aglycon (Figure 4) (Fischer et al., 2013; Grzegorzewski et al., 2011).

In a study conducted by Kovačević et al. (2016) on pomegranate juice treated with CP, no significant changes were observed in the case of color parameters. ΔE remained unchanged (*p < .05*), and also hue angle insignificantly decreased during the treatment. Even though a*, b*, and C* did not change for treated samples, they were lower than the untreated control sample. The control sample had the darkest color compared to those treated at 7 min with the brightest color. The results indicated that pomegranate color depends only on the gas flow rate, the processing time, and the sample volume, significantly affecting the juice color. They concluded that CP treatment positively affects the stability of anthocyanins in opaque pomegranate (Bursać Kovačević et al., 2016). In a study by Misra et al. (2014), strawberries were treated with cold atmospheric plasma (CAP) generated with a 60 kV dielectric barrier discharge. Results reported that L*, b*, and a* decreased insignificantly (Misra et al., 2014). As the results reported, a degradation in the color of dried wolfberry was observed in which L*, a*, and b* values of dried treated samples were higher than those of untreated control samples, in other words, 30–60 s of CP treatment resulted in a better color exterior outward than 15 s of processing time (Zhou et al., 2020).

As reported, the blueberry color is a complex characteristic of anthocyanin (Saftner et al., 2008). Atmospheric CP was applied to berries for 0, 15, 30, 45, 60, 90, or 120 s at a working distance of 7.5 cm using a mixture of 4 cubic feet per min (cfm) of CP jet and 7 cfm of ambient air. CP induced a darker and more blue surface color on blueberries; showing that CP treatment does not bleach the fruit, despite the significant loss of anthocyanins after 90 s of exposure. L*, a*, and b* surface color measurements were substantially affected after 120 s and 45 s, respectively (Lacombe et al., 2015). Results of CP treatment on anthocyanins and phenolic components in cherry juice showed that 3 min of treatment in the presence of nitrogen gas might cause an increase in anthocyanins and phenolic components in cherry juice and increase the intensity of the product color (Garofulić et al., 2015). Compared to the control sample, treated samples in their ideal conditions have the maximum amount of phenolic components, probably due to the concentration of tiny fragments by CP treatment. Results of a study in which atmospheric pressure CP was used for red chicory leaf decontamination indicated that 15 min of CP treatment did not affect the total color difference of the product. However, after 1 day of storage at 4°C, the product showed a desirable increase in dark red areas, and a significant reduction in incubated *E. coli* on the red chicory surface (Pasquali et al., 2016).

The results of CP treatment on the color parameters and anthocyanin content of the barberry juice showed that L*, b*, and a* values from treated barberry juice increase with the CP treatment time, representing the tendency of color to yellowness. It has been reported that despite a significant increase in lightness, redness, and yellowness of CP‐treated barberry juice, the color of barberry juice maintained in the consumer's expected spectrum. Thus, it can be concluded that CP treatment had no destructive effect on the product color (Rahnama & Abbaszade, 2018). In similar assumptions, the changes in pH should not be neglected. The highest amount of co‐pigmentation is observed at acidic pH of less than 3. This study showed that cold atmospheric gas plasma treatment causes a general color change in the product, even though it does not affect the intensity of the red color of the product (Gordillo et al., 2012).

#### 3.2.4 Betalains

Betalains are a class of pigments soluble in water and contain nitrogen in a heterocyclic form. They are further categorized into two subtypes, namely betacyanin, which appears as a red‐violet hue, and betaxanthin, which appears as a yellow‐orange hue. These entities are present in 13 plant families belonging to the Caryophyllales order and are typically characterized by their red appearance. In Caryophyllales, which are non‐containing anthocyanin plants, betalain has taken the place of anthocyanin and never found to co‐occur (Polturak & Aharoni, 2018; Stintzing & Carle, 2008).

The body of vascular plants is composed of three tissue systems, namely the dermal, the vascular, and the ground (or fundamental) tissues (Evert, 2006), which is a study betalain has been found in three of them all. Also, as mentioned in some studies, betalain accumulation has been found in vacuoles of reproductive and vegetative tissues, mainly subepidermal and epidermal tissues. In general, their presence in reproductive tissue such as petals, seeds, and fruits depends on the stages of plant growth, but it seems that their presence in vegetative tissue is controlled mainly by environmental conditions (Ramesh & Muthuraman, 2018; Yahia et al., 2011).

However, betalamic acid is known as the chromophore moiety; based on the ligands of betalamic acid, betaxanthin and betacyanin are the two classes of betalains. Betacyanin has higher wavelength absorption than betaxanthins (λ≈ 480 nm) due to the double bonds of the aromatic ring of cyclo‐dopa (a precursor for the pigment betalain in plants). The complex structure of betacyanin can be attributed to the occurrence of various substitutions, including glycosylation and acylation, in one or both 5‐6 carboxyl groups. Similarly, the formation of betaxanthin occurs in conjunction with amino acids, specifically the amino and amine groups, as well as the spontaneous incorporation of derivatives into the aldehyde group of betalamic acid. Moreover, condensation of betalamic acid and cyclo‐Dopa (cyclo‐3,4‐dihydroxyphenylalanine) forms betanidin, which is the key intermediate in betacyanin formation. In general, glycosylation of betanidin is accompanied by a hypsochromic shift of the resulting betacyanin, with glucose attached to C6 being less efficient than C5 glycosylation. While esterification with aliphatic acyl groups was reported to have little effect on the maximal absorption of betacyanin, acylation with aromatic acids results in a bathochromic shift (Esatbeyoglu et al., 2015; Esquivel, 2016; Khan & Giridhar, 2015).

However, various betalain degradation agents, including peroxidases, polyphenol oxidases, and glucosidase enzymes, as well as environmental factors such as O_2_, H_2_O_2_, high water activity, temperature, low glycosylation, and acylation degree, have been identified, betalain has been found to possess a broader pH stability range (pH = 3–7) in comparison to anthocyanins, which enables their utilization in low‐acidity food products (Azeredo, 2009; Khan, 2016; Stintzing & Carle, 2004).

##### How cold plasma affects betalain

**FIGURE 5:**
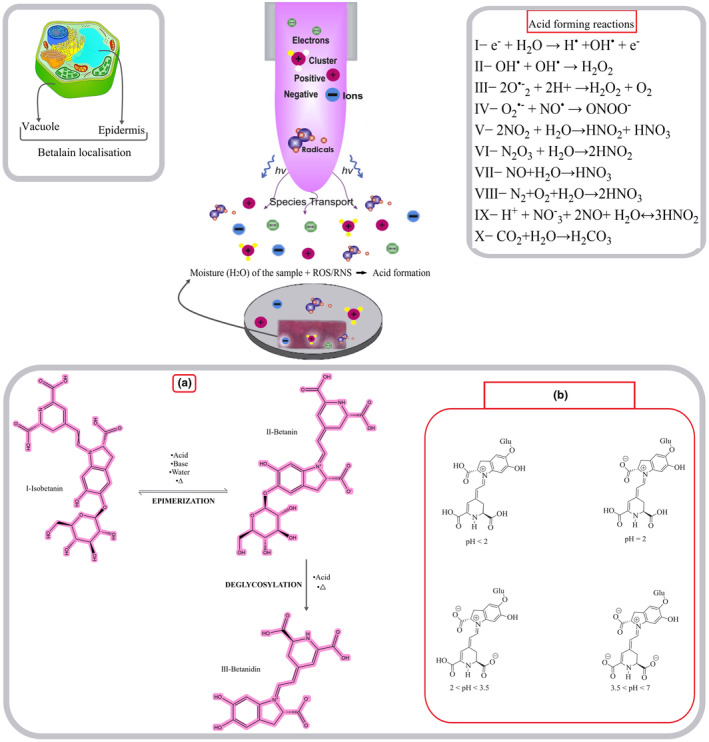
Effect of Cold Plasma on betalain. (a). Betanin alteration as a result of pH reduction. (b). Betalain structure changes in acidic pH induced by Cold plasma.

Since the reaction of reactive CP species with water has been discussed in detail in previous studies, when reactive (ROS/RNS) CP species, such as nitric oxide and nitrogen dioxide, react with water, acidic compounds will be formed because of this reaction. The dominant mechanism about the effect of CP on betalain pigment also follows this fact, where reactive species react with the moisture present on the surface of the food and produce acidic compounds, which causes medium pH reduction through acidic conditions. More examples about the formation of acidic compounds as a result of the reaction of CP active species with surface moisture are given in Figure 5 (Thirumdas et al., 2018; Varilla et al., 2020). Since betalain is stable in the pH range of 3–7, it has been determined that the decrease in pH changes the structure of betalain and causes deglycosylation and epimerization reactions. Acidification was found to induce recondensation of betalamic acid and cyclo Dopa 5‐O‐glucoside, which shifted the absorption maximum to a shorter wavelength (pH 2.0, 535 nm) and reduced absorption intensity. However, by increasing absorbance in the range of 575–650 nm, the hue of the solution changed from red to violet. It could be concluded that the recondensation of betalamic acid and cyclo Dopa 5‐O‐glucoside causes a change in hue from blue to violet (an increase in b*). This is how the CP, due to the reduction of pH by the formation of acidic compounds resulting from the reaction of the moisture of the food and active compounds, causes a betalain color shift from red to violet (Figure 5) (Calva‐Estrada et al., 2022; Devadiga & Ahipa, 2020; Fu et al., 2020).

Dzimitrowicz et al. (2021) reported that due to beetroot juice treated (BRJ) with FLA‐dc‐APGD (FLA‐direct current atmospheric pressure glow discharge) had the maximum ΔE, while BRJ treated with FLE‐pm‐rf‐APGD (FLA‐pulse‐modulated radiofrequency atmospheric pressure glow discharge) had the lowest. The result indicated that the corresponding increase in total phenolic compound concentration was not linked to observed changes in beetroot juice treated with FLA‐dc‐APGD. Accordingly, as seen by UV/Vis absorption, treated beetroot juice by FLC‐dc‐APGD‐ and FLE‐pm‐rf‐APGD was less red and greener. However, a* was slightly decreased and b* was significantly reduced. Due to the bathochromic effect, beetroot juice treated with FLA‐dc‐APGD appeared bluer and less yellow (Dzimitrowicz et al., 2021).

#### 3.2.5 Myoglobin

Myoglobin is an iron‐ and oxygen‐binding protein commonly found in the skeletal muscle tissue of vertebrates and nearly all mammals (Ordway & Garry, 2004). Myoglobin, like other goblins, is a member of the globin protein family and consists of eight alpha helices connected by loops. Myoglobin contains 153 amino acids and has an iron‐centered porphyrin ring. A proximal histidine group (His‐93) is directly bound to iron, while a distal histidine group (His‐64) remains near the opposite face. Although the distal imidazole is not bonded to the iron, it can interact with the O_2_ substrate. This interaction strengthens the O_2_ binding but not the carbon monoxide (CO) binding, which retains a 240‐fold greater affinity than O_2_. The binding of O_2_ causes substantial structural change at the iron center, which decreases in radius and relocates to the center of the N_4_ pocket. O_2_‐binding induces "spin‐pairing"; the five‐coordinate ferrous deoxy form has high spin, and the six‐coordinate oxy form has low spin and diamagnetic (Suman & Joseph, 2013). Myoglobin can take the forms of oxymyoglobin, carboxymyoglobin, and metmyoglobin, analogously to hemoglobin which takes the forms of oxyhemoglobin, carboxyhemoglobin, and methemoglobin. Experiments with the mitochondrial protease protection assay suggested that myoglobin localizes in the inner membrane of the mitochondria from the intermembrane space side. These findings strongly imply that myoglobin within the mitochondria of skeletal muscle may be involved in the regulation of mitochondrial respiration via complex IV (Koma et al., 2021).

##### 3.2.5.1 How cold plasma affects myoglobin

**FIGURE 6:**
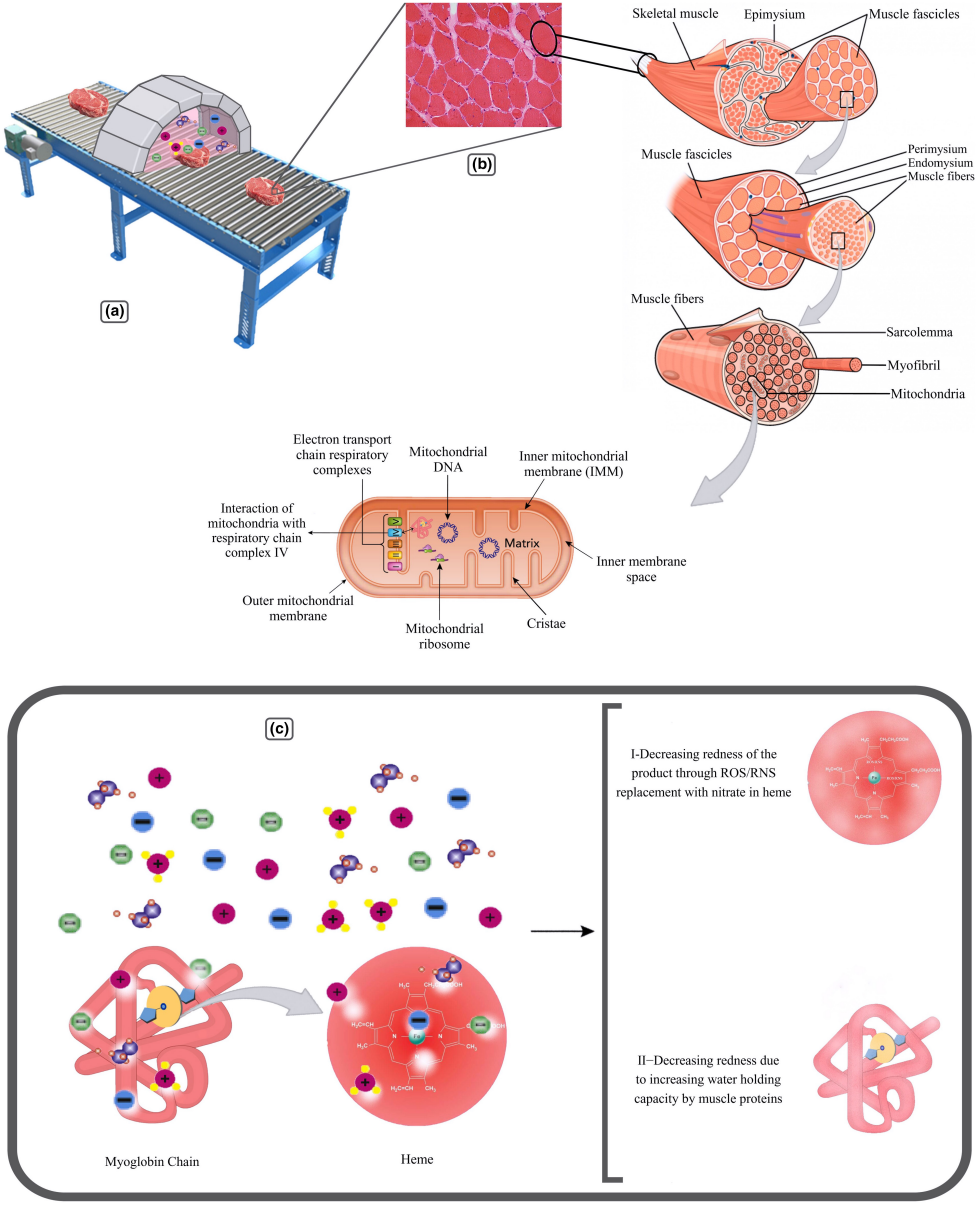
Effect of Cold Plasma on myoglobin. (a). Cold plasma treatment chamber. (b). MEAT sample texture. (c). Effect of ROS on myoglobin.

According to Zhang et al. (2022) findings, as the traditional chemical agent in meat (nitrite) is replaced by CP‐generated species, CP‐treated meat products are more vibrant red than untreated samples (Zhang et al., 2022). In addition, Koddy et al. (2021) reported that CP treatment increases the brightness value of hairtail samples due to the increase in hairtail muscle proteins' water‐holding capacity in treated samples (Figure 6) (Koddy et al., 2021). Moreover, it has been reported that color improvement of meats by giving a distinctive pinkish color has been achieved. This could be due to CP‐generated species like nitrogen species and reactive oxygen species (ROS) formations, such as nitrate (NO_3_) and nitrite (NO_2_). Furthermore, these reactive species cause the medium to become acidic, which follows a slight decrease in pH observed. Another possible mechanism of lower pH might be protein alteration by exposure to an acidic medium (Akhtar et al., 2022).

In limited studies, changing the color of animal‐origin food products in CP treatment has also been investigated. Slight changes were observed due to CP treatment in eggshells (Ragni et al., 2010; Vannini et al., 2009) and ready‐to‐eat Bresaola meat (Rød et al., 2012).

However, the a* and b* values of samples treated by DBD with helium‐oxygen working gas increased slightly, it was found that the L* value of bacon samples treated by CP decreased, possibly due to evaporation of its surface water (Kim et al., 2011). Regarding raw pork samples processed with CP, Fröhling et al. (2012) reported similar results; the L* and b* values significantly increased, while a* get decreased (Fröhling et al., 2012). The interaction between myoglobin and hydrogen peroxide and the formation of choleglobin or sulfhemoglobin as a result of the reaction between hydrogen sulfide and oxygen may account for the decrease in redness and a* value (Mancini & Hunt, 2005). Jayasena et al. (2015) observed that only a high exposure time (10 min) causes fat oxidation, while CP treatment for fresh pork and beef causes a slight decrease in product quality characteristics. Furthermore, colorimetric measurements indicated that a* decreases severely after 5 and 7.5 min. The DBD does not affect the L* value (Jayasena et al., 2015).

A study that investigated the effect of DBD atmospheric CP on qualitative and microbial properties of mackerel fish fillets showed no significant difference between a* and b* in the treated fish samples. However, the L* value or light intensity of treated samples decreased significantly. Therefore, it was concluded that CP treatment has no significant effect on the color of mackerel fish that can impress the customer's purchase (Albertos et al., 2017).

Yong et al. (2019) used CP treatment for processing the pork without sodium nitrate (Yong et al., 2019). For this purpose, processing parameters were used to achieve the desirable redness and color without adding chemical compounds of nitrate. These studies opened a new field in CP treatment; with this technology, new natural products without any chemical additives can be produced. Changing the color parameters of meat products could be due to the oxidation of pigments and fatty acids; due to the result of myoglobin reaction to O_3_ and other active species made from CP treatment, metmyoglobin increases. Sukarminah et al. (2017) reported that the presence of ROS and especially O_3_ causes myoglobin denaturation, loss of heme group, and emerging oxidation color, which results in the adverse color of products appearance (Sukarminah et al., 2017). Moreover, the Hanwood shredded beef pieces showed a reduction in redness and increased lightness when treated with O_3_ (Cho et al., 2014).

## 4 CONCLUSION

The industrial application of CP treatment in food processing is restricted because of insufficient knowledge and contradictory reports. This review aims to investigate the effectiveness of CP treatment as a novel and promising approach to improving the physicochemical properties of food products. CP has shown tremendous potential for the inactivation of diverse microorganisms, especially in fresh fruit and vegetables. The effects of CP treatment on the food colorants are influenced by various factors such as treatment time, gas type, CP power, electrode design, distance, structure, and molecular structure of pigments. The data in the reported studies suggested that further studies are required to understand the interaction of reactive CP species with natural food pigment. Regarding the unpleasant effects of CP on pigments, the optimized non‐thermal CP has a significant potential for food processing to produce products with acceptable color and appearance.

## AUTHOR CONTRIBUTIONS

**Yousef Ramezan:** Methodology (equal); project administration (equal); resources (equal); software (equal); supervision (equal); validation (equal); visualization (equal); writing – original draft (equal); writing – review and editing (equal). **Amir Kamkari:** Data curation (equal); formal analysis (equal); investigation (equal); methodology (equal); resources (equal); software (equal); validation (equal); writing – original draft (equal); writing – review and editing (equal). **Armita Lashkari:** Methodology (equal); software (equal); writing – original draft (equal). **Donya Moradi:** Investigation (equal); writing – original draft (equal); writing – review and editing (equal). **Abbas Najafi Tabrizi:** Writing – original draft (equal); writing – review and editing (equal).

## CONFLICT OF INTEREST STATEMENT

None.

## Data Availability

No data were used for the research described in the article.
